# Comparative analysis of inflammatory markers in HIV-positive individuals on antiretroviral therapy versus HIV-negative individuals in South Africa

**DOI:** 10.4102/ajlm.v14i1.2756

**Published:** 2025-01-26

**Authors:** Lungile Ndlovu, Nokukhanya Thembane, Ziningi N. Jaya

**Affiliations:** 1 Department of Biomedical Science, Faculty of Applied and Health Sciences, Mangosuthu University of Technology, Durban, South Africa

**Keywords:** inflammatory markers, antiretroviral therapy, ART, HIV-positive individuals, HIV-negative individuals, comparative analysis, immune response

## Abstract

**Background:**

HIV is associated with chronic inflammation and immune activation, which can persist even in individuals on antiretroviral therapy (ART), increasing the risk of cardiovascular disease, neurocognitive disorders, and other inflammatory conditions.

**Objective:**

This study comparatively investigates inflammatory markers among HIV-positive individuals receiving ART with those in HIV-negative individuals.

**Methods:**

We analysed retrospective laboratory results, including viral load, C-reactive protein (CRP), and D-dimer, from 275 individuals (aged 0–39 years) treated at a tertiary hospital in South Africa. The study period commenced on 02 December 2023 and ended on 28 October 2024. Spearman’s rank correlation was used to evaluate relationships among demographic factors, viral load, and inflammatory markers.

**Results:**

Findings revealed that HIV-positive individuals had significantly higher CRP levels (*r* = 0.140, *p* = 0.010), indicating persistent inflammation despite ART. D-dimer levels remained high within normal ranges across the sample, suggesting a generally low thrombotic risk, though elevated in a small subset of HIV-positive individuals. It also revealed that CRP levels were notably higher among male patients (*r* = 0.133, *p* = 0.014) compared to female patients. The age group with the highest inflammatory markers, such as CRP, were young adults (18–39 years old). Distribution results show the predominant gender being female (*n* = 211; 76.7%) versus male (*n* = 64; 23.3%).

**Conclusion:**

This study highlights the need for tailored strategies to manage inflammation and reduce cardiovascular risks in HIV-positive individuals, especially young adults and male patients.

**What this study adds:**

This study provides insight into specific inflammatory markers that are reduced or elevated in people living with HIV. It also assessed how ART influences the immune response in HIV-positive individuals, particularly in terms of inflammation. This can help in developing targeted therapies and monitoring disease progression.

## Introduction

KwaZulu-Natal, particularly Durban, has the highest HIV prevalence in South Africa, with approximately 27.3% of the population living with HIV.^1,2^ Of these, 61.8% are on antiretroviral therapy (ART), and 58.6% of ART users achieve viral suppression.^3^ Chronic immune activation and inflammation, which are central to HIV, contribute to complications such as neurocognitive disorders, coronary artery disease, liver/kidney issues, metabolic syndrome, osteoporosis, and certain cancers.^4^ This poses significant concerns for the South African Department of Health, as HIV is an inflammatory disease.^5^

Inflammation in HIV-positive individuals is indicated by elevated levels of biomarkers such as interleukin-6 (IL-6), C-reactive protein (CRP), and D-dimer.^6^ Raised CRP levels are linked to inflammation and a higher risk of cardiovascular disease,^7^ while increased IL-6 levels are associated with heart disease and cognitive decline.^8^ Elevated D-dimer levels signal enhanced coagulation and inflammation, increasing the risk of cardiovascular events and venous thromboembolism.^9^ Antiretroviral therapy has reduced HIV-related mortality and may lower inflammation, but also introduces other complications.^10^

A key consideration in managing HIV treatment is therapeutic drug monitoring, which can assess ART adherence and drug efficacy.^11^ Therapeutic drug monitoring is usually integrated to track ART drug concentrations in patients, and to determine how these levels correlate with inflammatory marker profiles (IL-6, CRP, D-dimer). This provides insight into whether suboptimal ART concentrations contribute to ongoing inflammation or inadequate viral suppression. While inflammatory markers in HIV-positive individuals have been studied, direct comparisons with HIV-negative individuals are limited.^12^ Thus, this study aims to address this gap by comparing IL-6, CRP, and D-dimer levels in both groups, helping to evaluate the effectiveness of ART in normalising inflammation. Monitoring these markers is essential for assessing treatment effectiveness and preventing complications, ultimately improving the health outcomes and quality of life for people living with HIV. This study aimed to investigate the distribution of inflammatory markers – CRP and D-dimer – and viral load among HIV-positive individuals in Durban, KwaZulu-Natal, and to explore potential correlations between these biomarkers. KwaZulu-Natal’s high HIV prevalence highlights ongoing challenges in managing HIV, particularly persistent inflammation, which contributes to complications such as cardiovascular diseases and neurocognitive decline.

## Methods

### Ethical considerations

Ethical approval for this study was granted by the Mangosuthu University of Technology Research Ethics Committee (reference number: RD5/28/2024). Retrospective laboratory data were obtained from the National Health Laboratory Service Academic Affairs and Research Management System. Patient details were anonymised to ensure confidentiality; no names, identity numbers, or any personal information was disclosed. Data were securely stored in a password-protected Microsoft Excel file, accessible only to the research team.

### Study design and setting

This study, conducted between 02 December 2023 and 28 October 2024, used a retrospective cohort design, in which retrospective laboratory results were utilised from an accredited laboratory based at a tertiary hospital in Durban, KwaZulu-Natal. KwaZulu-Natal is a region with one of the highest HIV prevalence rates in South Africa. The study analysed retrospective inflammatory marker laboratory results of people living with HIV and HIV-negative patients tested between January 2020 and December 2023. The data included inflammatory markers such as CRP, interleukins, and D-dimer.

### Data collection and processing

An Excel spreadsheet containing anonymised laboratory records was received. After obtaining the downloaded laboratory data from electronic records, a process of data cleaning and organisation was performed. Initially a total of 204 131 results were obtained for the requested period. The data set also included data not related to the study, leading to its removal and exclusion for the current study. After data cleaning, which saw the removal of duplicate patient results, and parameters ineligible for inclusion because of missing values, including interleukins, only 275 records were eligible for the study. Of these, 162 were HIV positive and 113 were HIV negative. Additionally, a new column was created from an existing variable, namely viral load, using the amputation technique to handle the missing data. The final analysis focused on comparing changes in inflammatory markers, such as CRP and D-dimer, with percentages, between HIV-positive individuals on ART and HIV-negative individuals.

Although this study utilised retrospective laboratory results, to the researcher’s knowledge the analysis of D-Dimer as an inflammatory marker was conducted using Sysmex CS-2100 automated blood coagulation analyser. The Cobas procedure for assessing CRP involves an immunoturbidimetric assay, a highly sensitive method used to quantify CRP levels in blood samples.

### Statistical analysis

The data were organised by gender, age, and inflammatory markers. Various charts were generated to visually represent the distribution of age, gender, and inflammatory markers such as CRP and D-dimer, with percentages in both HIV-positive and HIV-negative patients. Descriptive statistics such as mean, standard deviation, and mode, were calculated using Microsoft Excel. Spearman’s rank correlation coefficient was applied to assess the relationship between inflammatory markers and HIV status. Statistical analysis was conducted using descriptive tables and Spearman’s rank correlation for assessing the relationships between variables. The results of this analysis were then used to evaluate the hypothesis, ‘There is a significant positive correlation between viral load, CRP, and D-dimer levels in HIV-positive individuals ART compared to HIV-negative individuals’, and determine whether it could be rejected or supported.

## Results

### Demographic distribution

A total of 275 patient records were included in the study with 98.2% (*n* = 270) aged 18–39 years and 1.8% aged < 17 years. The majority of results included were from female patients (*n* = 211; 76.7%), and 23.3% (*n* = 64) from male patients (Table 1).

**TABLE 1:** Demographic distribution of study participants by age and gender in South Africa between January 2020 and December 2023.

Variable	Category	*n*	%
Age (years)	0–10	1	0.3
	11–17	4	1.5
	18–24	64	23.3
	25–30	67	24.4
	31–34	70	25.5
	35–39	69	25.1
Gender	Female	211	76.7
	Male	64	23.3

### Distribution of HIV infection by age group and gender among tested patients

A total of 113 (41%) results were HIV negative, while 162 (59%) were HIV positive. Among the 275 tested individuals, a higher prevalence of HIV was observed in the 18–39 years age group (158 positive cases; 57%), and among female patients (112 positive cases; 40%). The majority of participants aged < 17 years of age tested negative for HIV, with only four cases (1.5%) testing positive (Table 2).

**TABLE 2:** Distribution of HIV infection by age group and gender among tested patients in South Africa tested between January 2020 and December 2023.

Variable	Category	Total participants	HIV negative	HIV positive
Age group (years)	0–10	1	1	0
	11–17	4	0	4
	18–24	64	31	33
	25–30	67	29	38
	31–34	70	25	45
	35–39	69	28	41
Gender	Female	211	99	112
	Male	64	14	50

**Total**	**All participants**	**275**	**113**	**162**

### Distribution of viral load among tested patients by age group and gender

Table 3 shows the distribution of viral load among tested patients, categorised by age group and gender. It highlights the prevalence of undetectable viral loads, acceptable levels, and the need for monitoring or urgent medical attention across different subgroups.

**TABLE 3:** Distribution of viral load among tested patients by age group and gender in South Africa tested between January 2020 and December 2023.

Variable	Category	Total participants	Undetectable viral load		Acceptable levels (detectable)		Requires monitoring (very high)		Urgent medical attention needed	
		*N*	*n*	%	*n*	%	*n*	%	*n*	%
Age Group (years)	11–17	4	0	0.00	1	25.00	1	25.00	2	50.00
	18–24	33	28	84.85	4	12.12	1	3.03	0	0.00
	25–30	38	25	65.79	7	18.42	5	13.16	1	2.63
	31–35	45	31	68.89	10	22.22	3	6.67	1	2.22
	36–39	41	28	68.29	9	21.95	2	4.88	2	4.88
Gender	Female	211	99	49.92	70	33.17	11	5.21	31	14.69
	Male	64	14	21.88	25	39.06	5	7.81	19	29.69

### Distribution of normal and high C-reactive protein levels by age group and gender

Table 4 shows the distribution of normal and elevated CRP levels across different age groups and genders. Normal range used for children, adult male and female patients is less than 10.0 mg/L. The data reveal that individuals aged < 17 years predominantly had normal CRP levels, while the 18–39 years group had a nearly equal distribution of normal and high CRP levels. Additionally, female patients had a higher proportion of normal CRP levels, whereas male patients exhibited a greater incidence of elevated CRP levels.

**TABLE 4:** Distribution of normal and high C-reactive protein levels by age group and gender in South Africa tested between January 2020 and December 2023.

Variable	Category	Total participants	Normal CRP levels		High CRP levels	
		*N*	*n*	%	*n*	%
Age group (years)	0–17	5	4	80.00	1	20.00
	18–39	270	131	48.52	139	51.48
Gender	Female	211	117	55.45	94	44.55
	Male	64	18	28.13	46	71.88

**Total**	**All participants**	**275**	**135**	**49.09**	**140**	**50.91**

CRP, C-reactive protein.

### Distribution of D-dimer levels by age group and gender

Table 5 shows the distribution of normal and elevated D-dimer levels across age groups and genders. Reference normal range used was less than 0.50 mg/L. Most participants, regardless of age or gender, exhibited normal D-dimer levels, with only a small proportion showing elevated levels. Notably, all individuals in the < 17 years age group had normal levels, and elevated levels were rare among both female and male patients.

**TABLE 5:** Correlation among age, viral load, C-reactive protein, and D-dimer levels in South Africa tested January 2020 to December 2023.

Variable	Category	Total participants	Normal D-dimer levels		Elevated D-dimer levels	
		*N*	*n*	%	*n*	%
Age group (years)	0–17	5	5	100.00	0	0.00
	18–39	270	262	97.04	8	2.96
Gender	Female	211	204	96.69	7	3.31
	Male	64	63	98.44	1	1.56

**Total**	**All participants**	**275**	**267**	**97.09**	**8**	**2.91**

### Spearman’s rank correlation coefficients for age, viral load, C-reactive protein, and D-dimer levels

A weak negative correlation between age and viral load was observed, which was not statistically significant. However, a significant positive correlation was found between viral load and CRP (*r* = 0.140, *p* = 0.010), suggesting that higher viral loads may be linked to elevated CRP levels. Additionally, CRP and D-dimer levels exhibited a significant negative correlation (*r* = −0.133, *p* = 0.014), indicating that higher CRP levels might be associated with lower D-dimer levels (Table 6).

**TABLE 6:** Spearman’s rank correlation coefficients for age, viral load, C-reactive protein, and D-dimer levels (*N* = 275). South Africa, tested January 2020 to December 2023.

Variable	Category	Age	Viral load	C-reactive protein	D-dimer
Age	Correlation coefficient	1.000	-0.090	0.084	0.024
	Sig. (1-tailed)	-	0.068	0.082	0.349
	*N*	275	275	275	275
Viral load	Correlation coefficient	-0.090	1.000	0.140*	0.024
	Sig. (1-tailed)	0.068	-	0.010	0.345
	*N*	275	275	275	275
C-reactive protein	Correlation coefficient	0.084	0.140?	1.000	-0.133*
	Sig. (1-tailed)	0.082	0.010	-	0.014
	*N*	275	275	275	275
D-dimer	Correlation coefficient	0.024	0.024	-0.133*	1.000
	Sig. (1-tailed)	0.349	0.345	0.014	-
	*N*	275	275	275	275

* , correlation is significant at the 0.05 level (1-tailed).

## Discussion

This study aimed to investigate the distribution of inflammatory markers (including CRP and D-dimer), and viral load, among HIV-positive individuals in Durban, KwaZulu-Natal, and to explore potential correlations between these biomarkers. The study highlights that despite being on ART, there is a risk of elevated inflammatory markers (notably CRP). This suggests a need for better strategies to manage inflammation and reduce health risks.^4,5^

### Viral load and inflammation

The findings from this study demonstrate that, despite the majority of participants having low viral loads, a significant proportion of HIV-positive individuals still exhibited elevated CRP and D-dimer levels, suggesting ongoing inflammation despite adherence to ART.^13^ This observation is consistent with previous research highlighting the association of higher viral loads with increased immune activation and inflammation.^6^ A noteworthy finding in our study was the positive correlation between viral load and CRP levels (*r* = 0.140, *p* = 0.010), which aligns with prior research conducted in France (2012)^10^ and China (2023),^7^ and that suggests persistent immune activation in HIV-positive individuals, even under ART conditions. This finding supports the hypothesis that suboptimal viral suppression may contribute to sustained inflammation, which has been linked to various HIV-associated complications, including cardiovascular diseases and neurocognitive disorders.^7,8,14^

In contrast, the weak correlation observed between age and viral load, as well as age and CRP levels, suggests that other factors, such as ART adherence, immune status, and co-morbidities, may play a more prominent role in modulating viral load and inflammation in this cohort.^15,16^ The lack of a strong association between age and these biomarkers reflects the growing understanding that chronic inflammation in HIV-positive individuals can persist, regardless of age, particularly if viral suppression is not optimal.^10,17,18^ This finding highlights the importance of considering not only age but also other variables when assessing inflammation and the potential for HIV-related complications.

### C-reactive protein and D-dimer levels

The data from this study provide insights into the relationship between CRP, D-dimer, and viral load in HIV-positive individuals. Elevated CRP levels, indicative of systemic inflammation, were associated with higher viral loads, suggesting a link between HIV replication and immune activation. The negative correlation between CRP and D-dimer (*r* = −0.133, *p* = 0.014) is intriguing, as it contrasts with the typical positive association between inflammation and coagulation markers, such as D-dimer, in HIV.^9^ However, such findings are contradictory to findings of other studies, which have found a positive correlation between CRP and D-dimer and HIV-positive patients compared to negative controls.^19,20,21^ This inverse relationship may reflect a more complex inflammatory profile, influenced by factors such as ART efficacy, treatment duration, and individual variations in immune and coagulation responses.

While ART effectively reduces viral load and improves health outcomes, it may not fully resolve inflammation, particularly in cases of suboptimal viral suppression. Some studies suggest ART reduces CRP levels, but its effect on D-dimer remains more variable.^10^

### Gender differences

Our study also revealed notable gender-based differences in inflammatory markers, with female patients in the cohort exhibiting higher levels of normal CRP and lower rates of elevated CRP compared to male patients. This finding aligns with existing literature, which suggests that women tend to exhibit higher levels of immune activation and inflammatory responses than men.^12,22^ This gender-based disparity could be because of biological differences in immune function, as well as sociocultural factors that influence healthcare access and ART adherence. On the other hand, elevated D-dimer levels were more frequently observed in male patients, which is consistent with findings in a study conducted in Italy^23^ and a systematic review conducted in 2021,^9^ indicating that HIV-positive men may be at higher risk for thrombosis and other cardiovascular complications compared to women. This could suggest gender-specific risks for cardiovascular and thrombotic events among HIV-positive individuals, which may require tailored interventions to mitigate these risks.

### Implications for antiretroviral therapy and inflammation management

The persistence of elevated CRP and D-dimer levels despite ART use is a critical finding in this study. Although ART has revolutionised the management of HIV by reducing mortality and improving quality of life, it may not fully address the underlying inflammation that persists, even in individuals with viral suppression.^10,24,25^ These findings call attention to the need for ongoing monitoring of inflammatory markers, such as CRP and D-dimer, as part of routine clinical care for people living with HIV. Such monitoring could help identify individuals who are not experiencing adequate viral suppression, or those who may require adjustments to their ART regimen. Additionally, the results emphasise the potential value of therapeutic drug monitoring in the clinical practice of HIV management in different populations. Therapeutic drug monitoring could be used to assess ART adherence and its efficacy.

### Recommendations

Future research should explore the mechanisms driving inflammation in HIV-positive individuals, the role of personalised ART regimens in managing persistent inflammation, and the impact of ART on long-term health outcomes such as cardiovascular disease and cognitive decline. Future researchers should be more age specific and should include the length of ARV treatment. By advancing our understanding of the relationship between inflammation, HIV, and ART, this research has the potential to guide more targeted interventions aimed at improving the health and quality of life of people living with HIV, particularly in high-prevalence regions such as KwaZulu-Natal.

### Limitations of the study

The study had limitations. The relatively small sample size of 275 participants may limit the generalisability of the findings. A larger sample would provide more robust conclusions, especially when analysing demographic subgroups such as age and gender. The study focused solely on CRP and D-dimer as indicators of inflammation, which limits the findings. Including other markers, such as IL-6 or tumour necrosis factor-alpha, could offer a more comprehensive view of inflammation in HIV-positive individuals. Conducting the study in a single location may limit the applicability of the findings to other populations. Including different populations in other laboratories in South Africa would enhance the external validity of the results.

## Conclusion

In summary, this study highlights the complex interplay between viral load, inflammatory markers (CRP and D-dimer), and ART in HIV-positive individuals. While ART effectively suppresses the virus, it does not always normalise inflammation, as evidenced by the elevated CRP and D-dimer levels observed in this cohort. These findings suggest that HIV-related inflammation is a persistent issue, even in individuals with viral suppression. Given the potential link between chronic inflammation and HIV-related complications, ongoing monitoring of inflammatory biomarkers is essential for improving the management of HIV and reducing the risk of associated comorbidities.

## Data Availability

The authors confirm that the data supporting the findings of this study are available within the article.
